# Health-Related Quality of Life and Long-Term Survival After Cardiac Arrest

**DOI:** 10.1001/jamanetworkopen.2025.52832

**Published:** 2026-01-07

**Authors:** Emelie Dillenbeck, Per Nordberg, Akil Awad, Johan Israelsson, Araz Rawshani, Kristofer Årestedt, Anders Bremer, Jacob Hollenberg, Therese Djärv, Leif Svensson, Martin Jonsson

**Affiliations:** 1 Department of Clinical Science and Education, Center for Resuscitation Science, Södersjukhuset, Karolinska Institutet, Stockholm, Sweden; 2 Function Perioperative Medicine and Intensive Care, Karolinska University Hospital, Stockholm, Sweden; 3 Department of Physiology and Pharmacology, Karolinska Institutet, Stockholm, Sweden; 4 Department of Health and Caring Sciences, Faculty of Health and Life Sciences, Linnaeus University, Kalmar, Sweden; 5 Division of Cardiology, Department of Internal Medicine, Kalmar County Hospital, Kalmar, Sweden; 6 Department of Molecular and Clinical Medicine, Institute of Medicine, University of Gothenburg, Gothenburg, Sweden; 7 Department of Research, Region Kalmar County, Kalmar, Sweden; 8 Department of Medicine, Karolinska Institute, Stockholm, Sweden

## Abstract

**Importance:**

Health-related quality of life (HRQOL) follow-up can increase knowledge of cardiac arrest outcomes from the patient’s perspective. Whether HRQOL affects long-term survival is unknown.

**Objective:**

To investigate whether HRQOL 3 to 6 months after cardiac arrest is associated with long-term survival.

**Design, Setting, and Participants:**

This cohort study linked 5 national registers with nationwide coverage in Sweden across a 7-year period and included survivors of in-hospital cardiac arrest (IHCA) or out-of-hospital cardiac arrest (OHCA) between January 1, 2014, and December 31, 2019, with follow-up through June 30, 2021. Participants were adults surviving at least 90 days after IHCA or OHCA who completed follow-up EuroQoL 5-dimension 5-level (EQ-5D-5L) tool questionnaires. Analyses were performed December 2 to 20, 2024.

**Exposures:**

EQ-5D-5L level sum score (LSS; sum of EQ-5D-5L dimensions, ranging from 5 [no problems] to 25 [extreme problems in all dimensions]), and the visual analog scale EQ VAS, 3 to 6 months after cardiac arrest.

**Main Outcome and Measures:**

Long-term survival up to a maximum of 7 years among patients surviving IHCA and OHCA.

**Results:**

In total, 2000 survivors of IHCA (median [IQR] age, 73 [65-80] years, 66% male) and 1108 survivors of OHCA (median [IQR] age, 69 [59-75] years, 77% male) were included. There were 475 deaths among patients with IHCA and 132 deaths among patients with OHCA. For patients with IHCA, the LSS distribution was 394 (20%) for LSS 5, 1034 (52%) for LSS 6 to 10, and 572 (29%) for LSS 11 to 25. For patients with OHCA, the LSS distribution was 299 (27%) for LSS 5, 637 (58%) for LSS 6 to 10, and 168 (15%) for LSS 11 to 25. In the IHCA population, LSS 11 to 25 had higher hazard of death compared with LSS 5 (adjusted hazard ratio [AHR], 2.50 [95% CI, 1.82-3.43]), whereas LSS 6 to 10 did not (AHR, 1.21 [95% CI, 0.88-1.65]). In OHCA, no associations were found between LSS categories and long-term survival (LSS 11-25 vs LSS 5: AHR, 1.41 [95% CI, 0.83-2.42]; LSS 6-10 vs LSS 5: AHR, 0.88 [95% CI, 0.56-1.39]). In both IHCA and OHCA, spline modeling using LSS and EQ VAS as continuous variables showed significant increases in hazards of death with poorer HRQOL, although the estimates in the OHCA population showed greater uncertainty.

**Conclusions and Relevance:**

In this cohort study of patients who survived beyond 90 days after IHCA or OHCA, poorer HRQOL reported with EQ-5D-5L scores 3 to 6 months after cardiac arrest was associated with reduced long-term survival in both groups, with greater uncertainty for OHCA estimates. HRQOL assessment may help guide follow-up care.

## Key Points

**Question:**

Is health-related quality of life (HRQOL) 3 to 6 months after cardiac arrest associated with long-term survival?

**Findings:**

In this cohort study including 2000 survivors of in-hospital cardiac arrest and 1108 survivors of out-of-hospital cardiac arrest (OHCA), poorer HRQOL reported with the EuroQoL 5-dimension 5-level (EQ-5D-5L) tool questionnaire was associated with reduced long-term survival (assessed up to 7 years) in both cohorts, although uncertainty was higher among OHCA survivors.

**Meaning:**

Findings suggest that HRQOL measured after cardiac arrest with the EQ-5D-5L may help identify survivors at risk of reduced long-term survival and inform follow-up care and rehabilitation; further research should confirm clinical utility.

## Teaser

This cohort study examines whether health-related quality of life 3 to 6 months after cardiac arrest is associated with long-term survival among adults who survived in-hospital or out-of-hospital cardiac arrest.

## Introduction

Sudden cardiac arrest remains a major health challenge in the western world, with over 300 000 individuals experiencing out-of-hospital cardiac arrest (OHCA) in Europe annually, while the incidence of in-hospital cardiac arrest (IHCA) is more uncertain.^1,2^ Traditionally, outcomes after cardiac arrest have been reported in survival rates or by functional neurologic outcome scales. In recent years, however, there is growing interest in understanding the quality of life and long-term outcomes in cardiac arrest survivors.^3,4^

Health-Related Quality of Life (HRQOL) reflects an individual’s perception of how illness and treatments impact their daily life and functioning^4^ and provides a measure of outcomes after cardiac arrest from the patient’s perspective. The International Liaison Committee on Resuscitation^5,6,7^ and the Core Outcome Set for Cardiac Arrest group^3^ recommend HRQOL as a key outcome in cardiac arrest research, and international guidelines highlight the importance of follow-up on quality of life.^4,8^ In previous studies, patients surviving after cardiac arrest report an overall good HRQOL.^9,10,11,12,13,14^ However, many survivors experience cognitive, emotional, and physical problems, including fatigue,^15,16^ which can impact quality of life on an individual level. Interestingly, IHCA survivors have been shown to report significantly lower HRQOL levels compared with OHCA survivors.^10^ Other factors identified as being associated with worse self-reported HRQOL are female sex,^16,17^ neurological disabilities,^18^ and comorbidity.^17,19,20^

Prognostic factors associated with short-term survival after cardiac arrest are well documented. In contrast, long-term survival after cardiac arrest is not as well studied. However, several factors, such as neurologic recovery,^21^ returning to work,^22^ younger age,^23,24,25^ shockable rhythm,^24,26,27,28^ and bystander cardiopulmonary resuscitation^29^ have been identified as potential predictors of favorable long-term survival. In other cardiac populations, including patients with chronic heart failure and ischemic heart disease, findings suggest that HRQOL is associated with long-term survival.^30,31,32^ Additionally, 1 study found that OHCA survivors diagnosed with depression or anxiety had higher long-term mortality rates compared with those without these conditions.^33^ However, whether HRQOL among cardiac arrest survivors is associated with long-term survival is largely unknown. Clinically, if patients’ self-perceived health reflects their long-term prognosis, it could provide valuable insight for optimizing follow-up and rehabilitation strategies. To investigate this potential association, we used a large Swedish cohort of patients with cardiac arrest having structurally collected data on patient-reported outcomes. Our aim was to investigate whether the level of self-reported HRQOL 3 to 6 months after IHCA and OHCA is associated with long-term survival.

## Methods

### Study Design and Settings

This was a register-based cohort study including patients surviving IHCA and OHCA between January 1, 2014, and December 31, 2019, with follow-up until June 30, 2021. The study was conducted in Sweden, with a population of 10.3 million inhabitants in 2019.^34^ Data were obtained from the Swedish Register for Cardiopulmonary Resuscitation (SRCR), the Swedish Cause of Death Register, the Swedish National Patient Register, and Statistics Sweden. The study was approved by the Swedish Ethical Review Authority. The requirement of informed consent was waived, as it is not required in pseudoanonymized register-based research according to Swedish law. This report follows the Strengthening the Reporting of Observational Studies in Epidemiology (STROBE) reporting guideline.

### Population

Participants included in this study were patients with IHCA and emergency medical services (EMS)–treated patients with OHCA with a personal identity number registered in the SRCR. Patients were excluded if they were under 18 years of age, died within 90 days of cardiac arrest, experienced cardiac arrest in a region or hospital not performing HRQOL assessments during the study period, lacked a documented HRQOL assessment, or had a HRQOL assessment conducted by a proxy. IHCA and OHCA populations were analyzed separately.

### Data Sources

All Swedish residents have a unique personal identity number. This number enables linkage between registers.

#### SRCR

The SRCR is a national quality register with nationwide data on cardiac arrests. The register includes all patients experiencing cardiac arrest with attempted resuscitation, and data are collected in accordance with Utstein guidelines.^5,35^ Cardiac arrest characteristics were reported by the rapid response team (IHCA) or EMS personnel (OHCA). Other in-hospital data, including cerebral performance category (CPC) at discharge, were obtained from medical records by trained cardiopulmonary resuscitation (CPR) coordinators (specialist nurses).

Since 2013, a third registration is made 3 to 6 months after cardiac arrest, including assessments of HRQOL. For this registration, registry nurses manually screen patients who are alive 3 months after arrest to assess eligibility for HRQOL follow-up. Patients are excluded if they have severe cognitive dysfunction, language barriers (eg, aphasia or non-Swedish speakers), or decline participation. Eligible patients are sent questionnaires, including the EuroQoL 5-dimension 5-level (EQ-5D-5L) tool and the Hospital Anxiety and Depression Scale (HADS), along with an invitation letter for a telephone interview. During the interview, trained CPR coordinators, registry nurses, or cardiac rehabilitation nurses collect the responses to the questionnaires. Not all hospitals or regions perform this third registration. In 2014 to 2015, it was conducted by 59% of the hospitals and 67% of the regions for patients with IHCA and OHCA, respectively, increasing to 72% and 90%, respectively, in 2019.^36,37^

Patients alive after 3 months are informed of their inclusion in the register and the right to withdraw their participation at any time. The register has been validated and thoroughly described elsewhere.^38,39^

#### Other Data Sources

The date of death was obtained from The Swedish Cause of Death Register, which contains data on all deaths in Sweden since 1952.^40^ *International Statistical Classification of Diseases and Related Health Problems, Tenth Revision (ICD-10)* codes on comorbidities were collected from The National Patient Register, which contains *ICD-10* codes for all in-patient care in Sweden since 1987, and specialist outpatient visits since 2001.^41^ Both registers are maintained by the Swedish National Board of Health and Welfare. Data on disposable income and educational attainment were retrieved from the Longitudinal Integrated Database for Health Insurance and Labour Market Studies^42^ and data of birth region from the Total Population Register,^43^ both maintained by Statistics Sweden.^44^

### Exposure

The exposure in main analyses was self-reported health measured with the EQ-5D-5L questionnaire. Additional analyses were also performed using the HADS questionnaire as the exposure variable.

#### EQ-5D-5L

The EQ-5D-5L is a generic measure widely used for self-reported health measurement.^45^ It consists of 2 parts. The first part assesses health in 5 dimensions (mobility, self-care, usual activities, pain or discomfort, and anxiety or depression), with 5 response levels (no problem, slight problems, moderate problems, severe problems, and extreme problems).^45^ The responses can be summarized into a level sum score (LSS),^46,47^ ranging from 5 (no problems in any dimensions) to 25 (extreme problems in every dimension). In this study, the LSS was used both as a continuous variable and classified into 3 categories: LSS 5, LSS 6 to 10 and LSS 11 to 25. The second part consisted of the visual analog scale (EQ VAS) in which the responder rated the overall perceived health on the day of survey completion on a scale from 0 (worst imaginable health) to 100 (best imaginable health).^45^ The EQ-5D-5L is recommended as a core outcome in cardiac arrest research.^3^

#### HADS

HADS was developed to screen for symptoms of anxiety and depression in patients with somatic conditions. It consists of 2 subscales, HADS Anxiety and HADS Depression. Each subscale includes 7 items with 4 response categories, ranging from 0 to 3. The total score for each subscale ranges from 0 to 21, with higher scores indicating more severe symptoms. Various cutoff levels have been proposed,^48^ but for this study, continuous subscale scores were used. The HADS has been used in previous cardiac arrest studies^10^ and is recommended in international guidelines for screening of emotional problems.^8,49^

### Outcome

The primary outcome was long-term survival during the follow-up period (up to a maximum of 7 years) for OHCA and for IHCA. This outcome was used in all analyses.

### Statistical Analysis

Continuous variables are presented as medians and IQRs. Categorical data are presented as frequencies and percentages. Baseline balance was assessed using standardized mean differences (SMDs). A 2-sided *P* < .05 was considered statistically significant.

We imputed missing data on covariates using multiple imputation with chained equations (mice); 30 datasets were imputed and analyzed using the Rubin rules under the assumption that missing was at random. Survival time was measured from HRQOL follow-up date. We used a Cox proportional hazards regression model to analyze the association between survival and the 3-level categorization of the LSS, adjusting for sex, age, initial rhythm, year of cardiac arrest, county, disposable income, educational level, CPC at discharge, and comorbidities of cancer, heart failure, diabetes, ischemic heart disease, and kidney failure. For patients with IHCA, adjustments were also made for chronic obstructive pulmonary disease and cerebrovascular lesions. These were excluded from the OHCA model due to low prevalence (<5%) and minimal influence on the model. Adjusted survival curves were generated using the direct method via the adjustedCurves package in R.

Long-term survival was also analyzed with the LSS as a continuous variable using a Cox regression model with restricted cubic splines, adjusting for the same aforementioned covariates. The results were visualized in plots showing hazard ratios as functions of the LSS. Equivalent analyses were performed for the EQ VAS and the HADS Anxiety and Depression subscales.

To assess the association between each EQ-5D-5L dimension and long-term survival, we dichotomized the responses to each dimension into 2 groups: no problem or any problems. A Cox regression model was used, adjusting for the same aforementioned covariates. All statistical analyses were performed December 2 to 20, 2024 using R, version 4.2.2 (R Foundation for Statistical Computing).

## Results

### Patients and Baseline Characteristics

**Figure 1.**
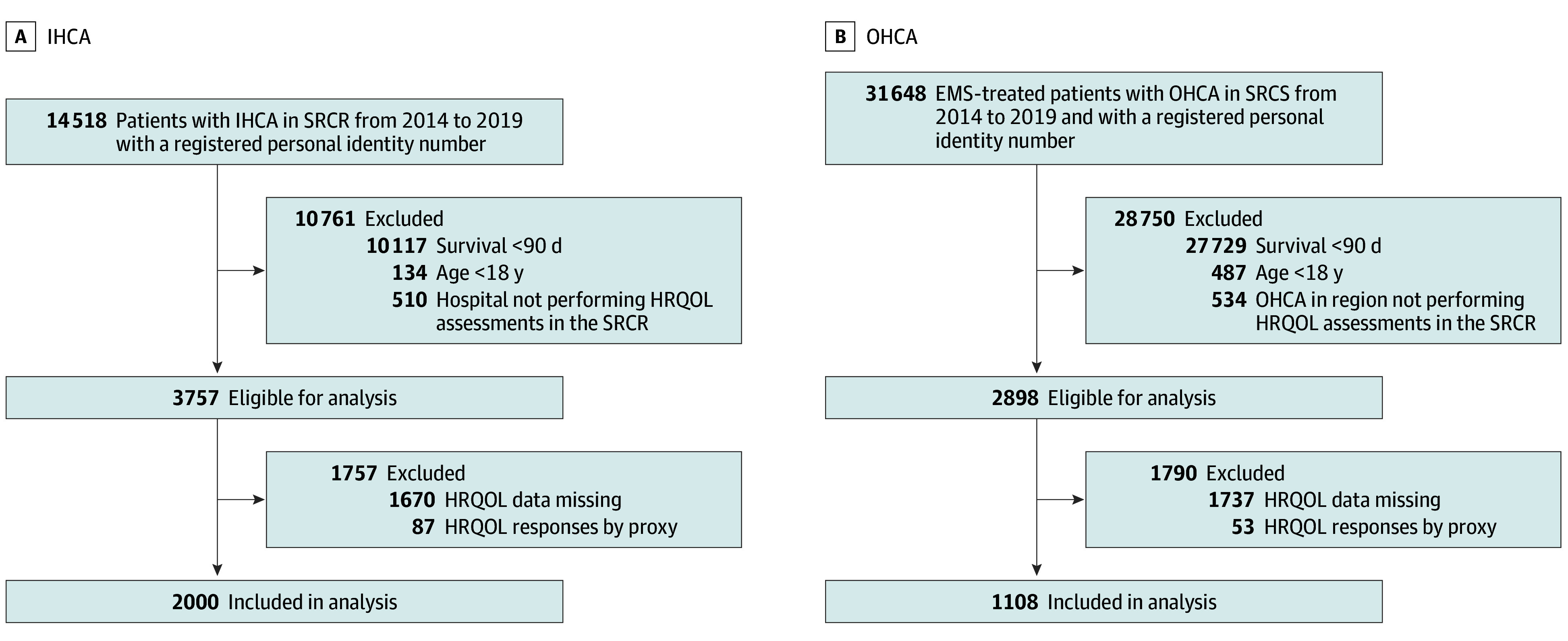
Flowchart of Included Patients EMS indicates emergency medical services; HRQOL, health-related quality of life; IHCA, in-hospital cardiac arrest; OHCA, out-of-hospital cardiac arrest; SRCR, Swedish Register for Cardiopulmonary Resuscitation.

During the study period, 14 518 patients with IHCA and 31 648 EMS-treated patients with OHCA were registered in the SRCR. After prespecified exclusions, 2000 patients with IHCA and 1108 patients with OHCA were included in the primary analysis. Among the exclusions, 1757 patients with IHCA (47%) and 1790 patients with OHCA (62%) were otherwise eligible but had missing HRQOL data or proxy responses (Figure 1).

Baseline characteristics for patients included in the primary analysis and those excluded for missing or proxy HRQOL are shown in the Table. Among included patients with IHCA, 688 of 2000 (34%) were female, 1312 (66%) were male, and their median [IQR] age was 73 [65-80] years. LSS was 5 for 394 patients (20%), 6 to 10 for 1034 patients (52%), and 11 to 25 for 572 patients (29%). CPC at discharge differed between included and excluded patients (overall SMD, 0.34 [95% CI, 0.27-0.41]; eg, for CPC 1: 1462 of 1740 patients [84%] vs 963 of 1346 patients [72%]). Among included patients with OHCA, 250 of 1108 (23%) were female, 858 of 1108 (77) were male, and their median [IQR] age was 69 [59-75] years. LSS was 5 in 299 of 1104 patients (27%), 6 to 10 in 637 of 1104 patients (58%), and 11 to 25 in 168 of 1104 patients (15%) (Table). CPC at discharge differed between included and excluded patients (overall SMD, 0.21 [95% CI, 0.13-0.29]; eg, for CPC 1: 847 of 1028 patients [82%] vs 883 of 1177 patients [75%]). Initial rhythm also differed between included and excluded patients (shockable: 862 of 998 patients (86%) vs 911 of 1344 patients (68%); SMD, 0.45 [95% CI, 0.37-0.54]).

**Table.** Baseline Characteristics of Included Patients and Patients Excluded Due to Missing HRQOL Data or Proxy Response

Characteristic	IHCA		SMD (95% CI)	OHCA		SMD (95% CI)
	Included in primary analysis (n = 2000)	Excluded from primary analysis (n = 1757)		Included in primary analysis (n = 1108)	Excluded from primary analysis (n = 1790)	
Sex, No./total No. (%)						
Female	688/2000 (34)	633/1757 (36)	0.03 (−0.03 to 0.10)	250/1108 (23)	505/1790 (28)	0.13 (0.06 to 0.21)
Male	1312/2000 (66)	1124/1757 (64)		858/1108 (77)	1285/1790 (72)	
Age, median (IQR), y	73 (65-80)	73 (62-82)	0.08 (0.01 to 0.14)	69 (59-75)	67 (53-77)	0.18 (0.10 to 0.25)
No.	2000	1753		1108	1775	
Witnessed cardiac arrest, No./total No. (%)	1877/1979 (95)	1621/1740 (93)	0.07 (0.01 to 0.14)	987/1099 (90)	1383/1755 (79)	0.31 (0.23 to 0.38)
Shockable initial rhythm, No./total No. (%)^a^	1071/1990 (54)	741/1747 (42)	0.23 (0.17 to 0.29)	862/998 (86)	911/1344 (68)	0.45 (0.37 to 0.54)
TTM of 32°C-36°C, No./total No. (%)^b^	133/1926 (7)	130/1623 (8)	0.04 (−0.02 to 0.11)	307/1064 (29)	329/1306 (25)	0.08 (0.00 to 0.16)
PCI, No./total No. (%)^b^	336/1061 (32)	204/866 (24)	0.18 (0.09 to 0.27	677/1093 (62)	570/1344 (42)	0.40 (0.32 to 0.48)
CABG, No./total No. (%)^b^	21/1060 (2)	14/863 (2)	0.03 (−0.06 to 0.12)	42/1085 (4)	45/1334 (3)	0.03 (−0.05 to 0.11)
ICD, No./total No. (%)^b^	50/599 (8)	35/544 (6)	0.07 (−0.04 to 0.19)	312/1056 (30)	270/1306 (21)	0.21 (0.12 to 0.29)
Region of birth, No./total No. (%)						
Europe	95/1983 (5)	130/1737 (8)	0.21 (0.14 to 0.27)	43/1107 (4)	92/1770 (5)	0.16 (0.08 to 0.23)
Nordic countries^c^	1814/1983 (91)	1476/1737 (85)		1036/1107 (94)	1584/1770 (89)	
Other	74/1983 (4)	131/1737 (8)		28/1107 (2)	94/1770 (5)	
Educational level, No./total No. (%)						
Primary	634/1982 (32)	598/1712 (35)	0.07 (0.00 to 0.13)	301/1102 (27)	581/1737 (33)	0.17 (0.09 to 0.24)
Secondary ≤2 y	879/1982 (44)	710/1712 (41)		487/1102 (44)	770/1737 (44)	
Postsecondary >2 y	469/1982 (24)	404/1712 (24)		314/1102 (28)	389/1737 (22)	
Income, median (IQR)^d^	182 (146 to 267)	162 (135 to 224)	0.15 (0.09 to 0.21)	212 (158 to 329)	172 (137 to 252)	0.10 (0.02 to 0.17)
No.	2000	1753		1108	1775	
Comorbidities, No./total No. (%)^e^						
COPD	182/2000 (9)	169/1757 (10)	−0.02 (−0.08 to 0.05)	38/1108 (3)	115/1790 (6)	−0.14 (−0.21 to −0.06)
IHD	843/2000 (42)	691/1757 (39)	0.06 (−0.01 to 0.12)	357/1108 (32)	509/1790 (28)	0.08 (0.01 to 0.16)
Heart failure	438/2000 (22)	399/1757 (23)	−0.02 (−0.08 to 0.04)	176/1108 (16)	264/1790 (15)	0.03 (−0.04 to 0.11)
Cerebrovascular lesion	134/2000 (7)	193/1757 (11)	−0.15 (−0.22 to −0.09)	46/1108 (4)	134/1790 (8)	−0.14 (−0.22 to −0.07)
Diabetes	418/2000 (21)	408/1757 (23)	−0.06 (−0.12 to 0.01)	142/1108 (13)	222/1790 (12)	0.01 (−0.06 to 0.09)
Hypertension	1082/2000 (54)	901/1757 (51)	0.06 (−0.01 to 0.12)	361/1108 (33)	640/1790 (36)	−0.07 (−0.14 to 0.01)
Kidney failure	222/2000 (11)	244/1757 (14)	−0.08 (−0.15 to −0.02)	53/1108 (5)	109/1790 (6)	−0.06 (−0.13 to 0.02)
Cancer	465/2000 (23)	395/1757 (22)	0.02 (−0.05 to 0.08)	155/1108 (14)	267/1790 (15)	−0.03 (−0.10 to 0.05)
Dementia	43/2000 (2)	105/1757 (6)	−0.19 (−0.26 to −0.13)	12/1108 (1)	73/1790 (4)	−0.19 (−0.26 to −0.11)
CPC at discharge, No./total No. (%)						
1	1462/1740 (84)	963/1346 (72)	0.34 (0.27 to 0.41)	847/1028 (82)	883/1177 (75)	0.21 (0.13 to 0.29)
2	201/1740 (12)	215/1346 (16)		140/1028 (14)	199/1177 (17)	
3	71/1740 (4)	159/1346 (12)		37/1028 (4)	78/1181 (7)	
4	6/1740 (<1)	9/1346 (<1)		4/1028 (<1)	17/1177 (1)	
EQ-5D-5L LSS, median (IQR)	8.0 (6.0 to 11.0)	NA	NA	7.0 (5.0 to 9.0)	NA	NA
No.	2000			1104		
EQ-5D-5L LSS 5, No./total No. (%)	394/2000 (20)	NA	NA	299/1104 (27)	NA	NA
EQ-5D-5L LSS 6-10, No./total No. (%)	1034/2000 (52)	NA	NA	637/1104 (58)	NA	NA
EQ-5D-5L LSS 11-25, No./total No. (%)	572/2000 (29)	NA	NA	168/1104 (15)	NA	NA
EQ VAS score, median (IQR)	70 (50 to 80)	NA	NA	80 (65 to 90)	NA	NA
No.	1998	NA	NA	1108	NA	NA
HADS Anxiety score, median (IQR)	2.0 (0.0 to 6.0)	NA	NA	2.0 (0.0 to 5.0)	NA	NA
No.	1994	NA	NA	1107	NA	NA
HADS Depression score, median (IQR)	2.0 (1.0 to 5.0)	NA	NA	2.0 (0.0 to 4.0)	NA	NA
No.	1993	NA	NA	1106	NA	NA

Abbreviations: CABG, coronary artery bypass graft; COPD, chronic obstructive pulmonary disease; CPC, cerebral performance category; EQ-5D-5L, EuroQoL 5-dimension 5-level tool; HADS, Hospital Anxiety and Depression Scale; HRQOL, health-related quality of life; ICD, implantable cardioverter defibrillator; IHCA, in-hospital cardiac arrest; IHD, ischemic heart disease; LSS, level sum score; NA, not applicable; OHCA, out-of-hospital cardiac arrest; PCI, percutaneous coronary intervention; SMD, standardized mean difference; TTM, targeted temperature management.

^a^ Ventricular fibrillation or pulseless ventricular tachycardia.

^b^ During hospitalization for cardiac arrest.

^c^ Nordic countries include Sweden, Norway, Denmark, and Iceland.

^d^ Mean disposable yearly income during 10 years before cardiac arrest, in thousands of Swedish kronor (1 Swedish kronor is approximately US $0.11).

^e^ Diagnosed up to 90 days after cardiac arrest.

### Outcomes

**Figure 2.**
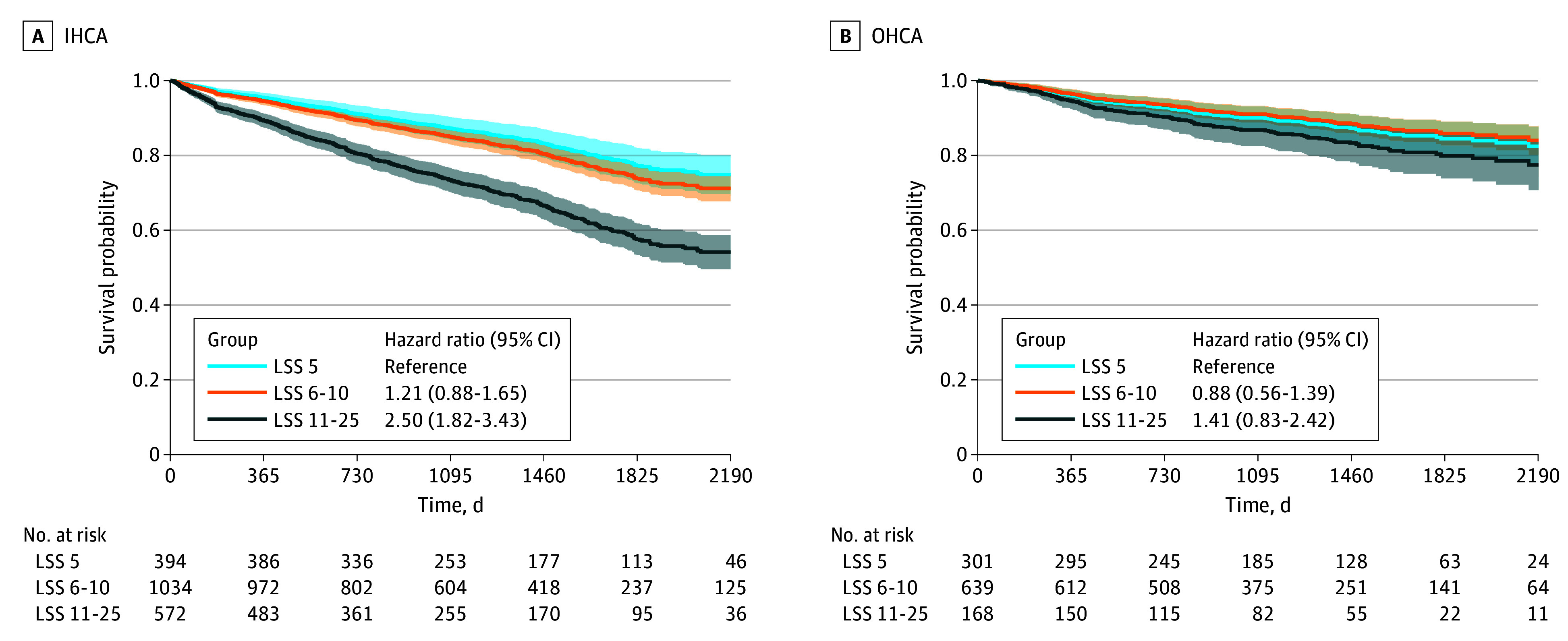
Adjusted Survival Curves for In-Hospital Cardiac Arrest (IHCA) and Out-of-Hospital Cardiac Arrest (OHCA) by EuroQoL 5-Dimension 5-Level Tool Level Sum Score (LSS) Categories Lines represent adjusted hazard ratios, with 95% CIs shown in shading, for each group.

**Figure 3.**
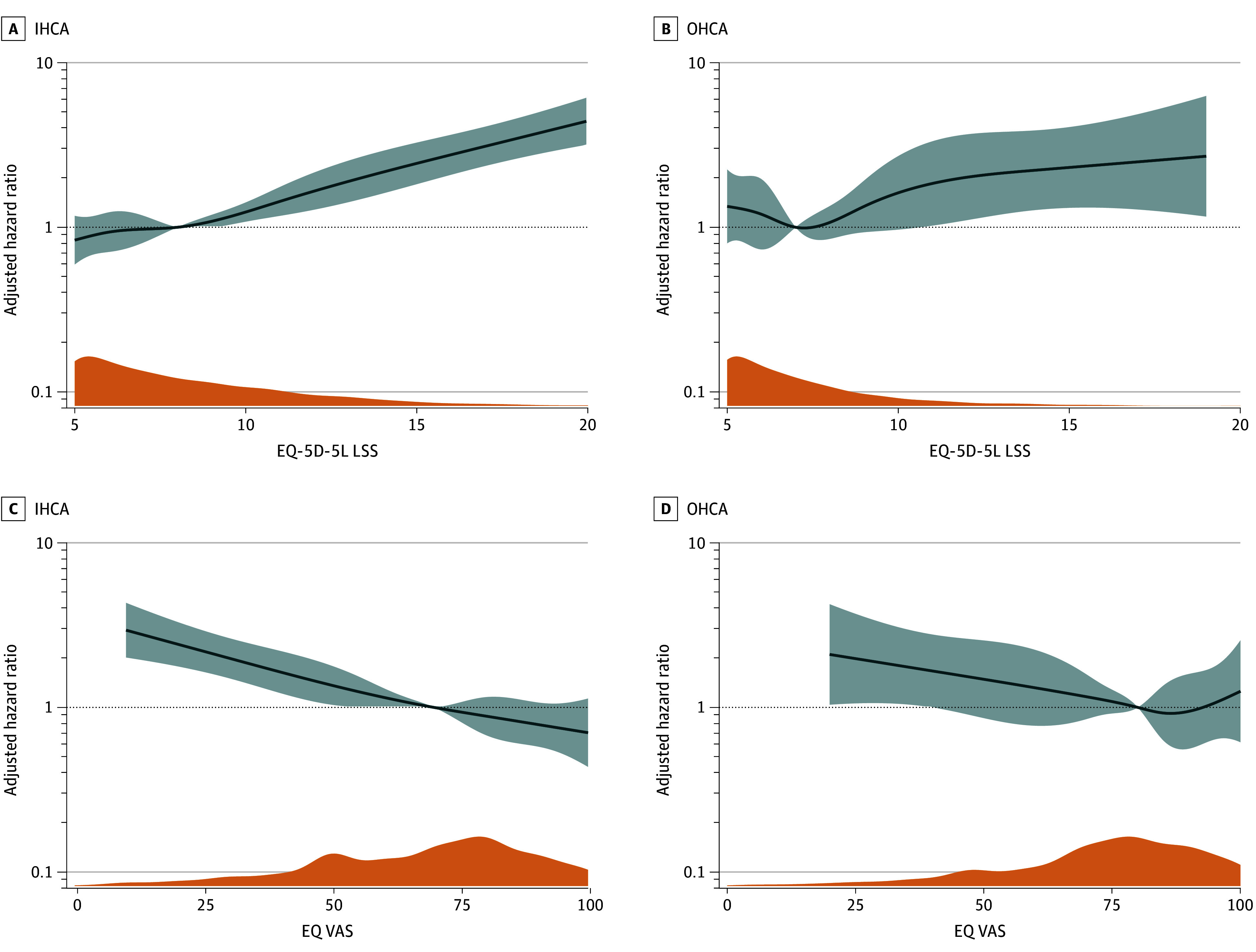
Adjusted Hazard Ratios for Death During Follow-Up as Functions of EuroQoL 5-Dimension 5-Level (EQ-5D-5L) Tool Level Sum Score (LSS) and the Visual Analog Scale EQ VAS Score Dark blue lines represent adjusted hazard ratios, with 95% CIs shown in lighter blue shading. Orange plots show the density of either EQ-5D-5L LSS or EQ VAS scores. IHCA indicates in-hospital cardiac arrest; OHCA, out-of-hospital cardiac arrest.

**Figure 4.**
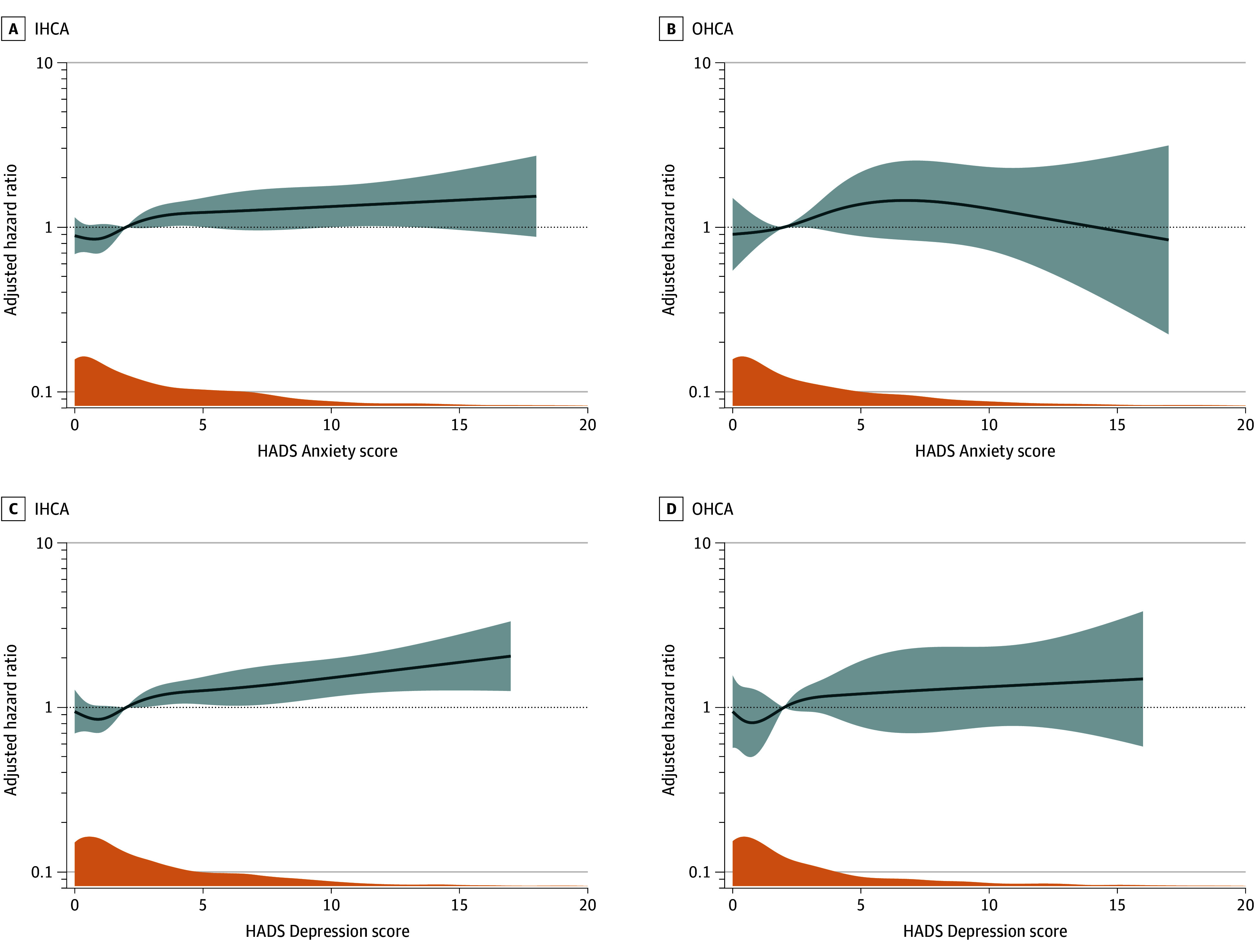
Adjusted Hazard Ratios for Death During Follow-Up as Functions of Hospital Anxiety and Depression Scale (HADS) Anxiety and Depression Scores Dark blue lines represent adjusted hazard ratios, with 95% CI shown in lighter blue shading. Orange plots show the density of either HADS Anxiety scores or HADS Depression scores. IHCA indicates in-hospital cardiac arrest; OHCA, out-of-hospital cardiac arrest.

During the study period, 475 deaths (24%) occurred in the IHCA population and 132 deaths (12%) in the OHCA population. Patients with IHCA reporting the poorest HRQOL (LSS 11-25) had significantly reduced long-term survival compared with those reporting no problems (LSS 5), with an adjusted hazard ratio (AHR) for death during follow-up of 2.50 (95% CI, 1.82-3.43). No association was observed for the LSS 6 to 10 group compared with LSS 5 (AHR, 1.21 [95% CI, 0.88-1.65]) (Figure 2). In the OHCA population, no associations were found between LSS categories and long-term survival (for LSS 6-10: AHR, 0.88 [95% CI, 0.56-1.39]; for LSS 11-25: AHR, 1.41 [95% CI, 0.83-2.42]). Figure 2 presents the adjusted survival curves, and crude curves are available in eFigures 1 and 2 in Supplement 1. In both IHCA and OHCA populations, spline modeling using LSS and EQ VAS as continuous variables showed significant increases in hazards of death with poorer HRQOL, although the estimates in the OHCA population showed greater uncertainty (Figure 3). The HADS Depression score was associated with long-term survival in IHCA, whereas no associations were found in the OHCA population or for HADS Anxiety scores (Figure 4). eFigures 3 and 4 in Supplement 1 show the unadjusted plots corresponding to Figures 3 and 4.

In the IHCA population, reporting problems in any of the individual EQ-5D-5L domains was independently associated with increased hazard of death during follow-up (eFigure 5 in Supplement 1). In the OHCA group, reporting problems with self-care or usual activities, but not the other domains, was independently associated with increased hazard of death (eFigure 5 in Supplement 1).

## Discussion

This Swedish register-based cohort study including patients who survived beyond 90 days after IHCA or OHCA and completed follow-up HRQOL questionnaires at 3 to 6 months after arrest found that poorer HRQOL as measured with EQ-5D-5L LSS and EQ VAS scores was associated with significantly reduced long-term survival among patients with IHCA. The same association was found among patients with OHCA, but estimates showed greater uncertainty. Depressive symptoms according to HADS scores were associated with reduced long-term survival among patients with IHCA, whereas no such association was found among patients with OHCA. In addition, there was no association between anxiety symptoms as assessed with HADS scores and long-term survival for either group.

Our findings that EQ-5D LSS and VAS scores were associated with long-term survival are consistent with previous studies in other cardiac populations.^30,31,32^ Similar associations have also been found in these populations using alternative HRQOL instruments.^50,51^ These findings suggest that, alongside more objective factors, the patient’s own perception of how their illness and treatment impact their quality of life could potentially serve as a prognostic factor for long-term survival. These insights may be valuable for clinicians during long-term follow-up, as patients reporting poor HRQOL may warrant closer monitoring and optimized rehabilitation directed at underlying health issues. However, further prospective studies are needed to investigate whether HRQOL-guided interventions alter outcomes.

The association between long-term survival and EQ-5D-5L scores may have several explanations. For example, comorbidities are associated with reporting poorer HRQOL,^17,19,20^ which in turn may influence survival.^19,28^ However, HRQOL also reflects the overall burden of illness, including symptoms, disability, and functional limitations that may not be fully captured in clinical assessments. In our study as well as others,^31,52^ adjusting for comorbidities resulted in some attenuation of the hazards, yet significant findings remained, indicating that the EQ-5D-5L provides additional, clinically relevant information. A similar pattern was observed after adjusting for neurological function. Moreover, the EQ-5D-5L integrates aspects of physical, mental, and social health, which may influence behaviors such as physical activity, treatment adherence, and health care seeking and thereby longevity. The EQ-5D-5L may also reflect frailty and functional reserve, which are well-established predictors of mortality.^53^ Future research using more detailed measures of HRQOL and data on care after hospital discharge are needed to better understand the mechanisms underlying these associations.

We found no association between LSS categories and long-term survival in the OHCA population, although the pattern was similar to the findings among patients with IHCA. Spline modeling of continuous LSS and EQ-VAS scores did show associations with long-term survival, but with greater uncertainty than among patients with IHCA. One previous study has investigated this association among survivors of OHCA and found similar results.^22^ Compared with patients with IHCA, patients with OHCA were younger, had fewer comorbidities, and better HRQOL as measured with the EQ-5D-5L. Missing HRQOL data was also more common in the OHCA population (62% vs 47%), introducing greater selection bias. Follow-up mortality was lower (12% vs 24%), which may have limited power. It remains unclear whether the greater uncertainty in the OHCA population was due to a sample size too small, better overall HRQOL, selection bias, or too short follow-up period limiting the number of observed deaths—or simply a weaker association. Further studies with larger cohorts and extended follow-up periods are needed to investigate this.

In our study, we found an association between HADS Depression subscale scores and long-term survival among survivors of IHCA but not among those with OHCA. HADS Anxiety subscale scores did not show any associations with long-term survival in either group. Interestingly, a study conducted in South Korea reported higher long-term mortality among survivors of OHCA diagnosed with anxiety or depression.^33^ In other cardiac populations, symptoms of depression and anxiety have been associated with increased mortality, but findings for anxiety are more inconsistent.^54,55,56^ Reported prevalence rates of anxiety and depression symptoms among survivors of cardiac arrest range from 15% to 34% and 13% to 20%, respectively,^12,14,57,58^ with our findings aligned with the lower end. However, individuals with more severe psychological distress may have been less likely to complete the HRQOL assessments, leading to their underrepresentation in our cohort and potentially influencing the results.

### Strengths and Limitations

The major strength of this study is the large, nationwide cohorts of IHCA and OHCA survivors, representing one of the largest datasets of its kind. The findings provide a strong rationale for further prospective studies.

This study has several limitations. Most notably, HRQOL data were missing for 62% of patients with OHCA and 47% of patients with IHCA who were otherwise eligible. Completing HRQOL questionnaires requires adequate cognitive and physical function—an inherent limitation shared with other studies in this field, and patients with the most severe impairments are thereby systematically excluded. This limitation introduces selection bias, and the findings may not be generalizable to patients with the most severe impairments. The exclusion of individuals with language barriers from HRQOL assessments may also limit generalizability. Another limitation is that both the EQ-5D-5L and HADS are generic instruments. Although widely used in cardiac arrest populations, they may not fully capture key aspects specific to survivors of cardiac arrest, such as cognitive impairment, fatigue, and social reintegration. This limitation may affect the precision of all HRQOL research in cardiac arrest. Currently, no cardiac arrest–specific HRQOL tool exists, although development efforts are under way.^45^ Additionally, HRQOL may vary over time. Although the 3 to 6–month follow-up window used in this study aligns with current recommendations for postcardiac arrest HRQOL assessment,^3,6,7^ it may not represent the optimal time point for long-term prognostication, potentially misclassifying the exposure and biasing associations toward the null. Furthermore, baseline HRQOL assessment prior to cardiac arrest was not available, limiting our ability to assess changes over time. Additionally, the observational design of the study cannot exclude the potential of residual confounding, and the results should be interpreted as such.

## Conclusions

In this Swedish cohort study of patients who survived beyond 90 days after IHCA or OHCA, poorer HRQOL reported with the EQ-5D-5L at 3 to 6 months after arrest was associated with reduced long-term survival in both groups, with greater uncertainty in the OHCA estimates. Symptoms of depression, as measured using HADS scores, were associated with reduced long-term survival among patients with IHCA; no such association was found among patients with OHCA or for symptoms of anxiety. Future studies are needed to investigate whether individuals reporting poor HRQOL may benefit from targeted follow-up and rehabilitation.

## Notes

Supplement 1. **eFigure 1.** Unadjusted survival plot for patients with in-hospital cardiac arrest (IHCA) **eFigure 2.** Unadjusted survival plot for patients with out-of-hospital cardiac arrest (OHCA) **eFigure 3.** Unadjusted hazard ratios (HRs) for death during follow-up as functions of EQ-5D-5L level sum score (LSS) and EQ VAS in IHCA and OHCA populations **eFigure 4.** Unadjusted hazard ratios (HRs) for death during follow-up as functions of Hospital anxiety and depression scale (HADS) anxiety scores and HADS depression scores in IHCA and OHCA populations **eFigure 5.** Forest plot of adjusted hazards ratios (HRs) for death during study follow-up for each EQ dimension in IHCA and OHCA populations

Supplement 2.
Data Sharing Statement

## References

[1] Empana JP, Lerner I, Valentin E, ; ESCAPE-NET Investigators. Incidence of sudden cardiac death in the European Union. J Am Coll Cardiol. 2022;79(18):1818-1827. doi:10.1016/j.jacc.2022.02.041 35512862

[2] Gräsner JT, Herlitz J, Tjelmeland IBM, . European Resuscitation Council Guidelines 2021: epidemiology of cardiac arrest in Europe. Resuscitation. 2021;161:61-79. doi:10.1016/j.resuscitation.2021.02.007 33773833

[3] Haywood K, Whitehead L, Nadkarni VM, ; COSCA Collaborators. COSCA (Core Outcome Set for Cardiac Arrest) in Adults: an advisory statement from the International Liaison Committee on Resuscitation. Resuscitation. 2018;127:147-163. doi:10.1016/j.resuscitation.2018.03.022 29706235

[4] Sawyer KN, Camp-Rogers TR, Kotini-Shah P, ; American Heart Association Emergency Cardiovascular Care Committee; Council on Cardiovascular and Stroke Nursing; Council on Genomic and Precision Medicine; Council on Quality of Care and Outcomes Research; and Stroke Council. Sudden cardiac arrest survivorship: a scientific statement from the American Heart Association. Circulation. 2020;141(12):e654-e685. doi:10.1161/CIR.0000000000000747 32078390

[5] Perkins GD, Jacobs IG, Nadkarni VM, ; Utstein Collaborators. Cardiac arrest and cardiopulmonary resuscitation outcome reports: update of the Utstein Resuscitation Registry Templates for Out-of-Hospital Cardiac Arrest: a statement for healthcare professionals from a task force of the International Liaison Committee on Resuscitation (American Heart Association, European Resuscitation Council, Australian and New Zealand Council on Resuscitation, Heart and Stroke Foundation of Canada, InterAmerican Heart Foundation, Resuscitation Council of Southern Africa, Resuscitation Council of Asia); and the American Heart Association Emergency Cardiovascular Care Committee and the Council on Cardiopulmonary, Critical Care, Perioperative and Resuscitation. Circulation. 2015;132(13):1286-1300. doi:10.1161/CIR.0000000000000144 25391522

[6] Bray JE, Grasner JT, Nolan JP, ; International Liaison Committee on Resuscitation. Cardiac arrest and cardiopulmonary resuscitation outcome reports: 2024 update of the Utstein Out-of-Hospital Cardiac Arrest Registry Template. Circulation. 2024;150(9):e203-e223. doi:10.1161/CIR.0000000000001243 39045706

[7] Nolan JP, Berg RA, Andersen LW, . Cardiac arrest and cardiopulmonary resuscitation outcome reports: update of the Utstein Resuscitation Registry Template for In-Hospital Cardiac Arrest: a consensus report from a task force of the International Liaison Committee on Resuscitation (American Heart Association, European Resuscitation Council, Australian and New Zealand Council on Resuscitation, Heart and Stroke Foundation of Canada, InterAmerican Heart Foundation, Resuscitation Council of Southern Africa, Resuscitation Council of Asia). Circulation. 2019;140(18):e746-e757. doi:10.1161/CIR.0000000000000710 31522544

[8] Nolan JP, Sandroni C, Böttiger BW, . European Resuscitation Council and European Society of Intensive Care Medicine guidelines 2021: post-resuscitation care. Intensive Care Med. 2021;47(4):369-421. doi:10.1007/s00134-021-06368-4 33765189 PMC7993077

[9] Smith K, Andrew E, Lijovic M, Nehme Z, Bernard S. Quality of life and functional outcomes 12 months after out-of-hospital cardiac arrest. Circulation. 2015;131(2):174-181. doi:10.1161/CIRCULATIONAHA.114.011200 25355914

[10] Djärv T, Bremer A, Herlitz J, . Health-related quality of life after surviving an out-of-hospital compared to an in-hospital cardiac arrest: a Swedish population-based registry study. Resuscitation. 2020;151:77-84. doi:10.1016/j.resuscitation.2020.04.002 32294490

[11] Tiainen M, Vaahersalo J, Skrifvars MB, Hästbacka J, Grönlund J, Pettilä V. Surviving out-of-hospital cardiac arrest: the neurological and functional outcome and health-related quality of life one year later. Resuscitation. 2018;129:19-23. doi:10.1016/j.resuscitation.2018.05.011 29775641

[12] Yonis H, Sørensen KK, Bøggild H, . Long-term quality of life after out-of-hospital cardiac arrest. JAMA Cardiol. 2023;8(11):1022-1030. doi:10.1001/jamacardio.2023.2934 37703007 PMC10500433

[13] Wimmer H, Lundqvist C, Šaltytė Benth J, . Health-related quality of life after out-of-hospital cardiac arrest—a five-year follow-up study. Resuscitation. 2021;162:372-380. doi:10.1016/j.resuscitation.2021.01.036 33571604

[14] Israelsson J, Bremer A, Herlitz J, . Health status and psychological distress among in-hospital cardiac arrest survivors in relation to gender. Resuscitation. 2017;114:27-33. doi:10.1016/j.resuscitation.2017.02.006 28216089

[15] Moulaert VRM, van Heugten CM, Gorgels TPM, Wade DT, Verbunt JA. Long-term outcome after survival of a cardiac arrest: a prospective longitudinal cohort study. Neurorehabil Neural Repair. 2017;31(6):530-539. doi:10.1177/1545968317697032 28506147

[16] Bohm M, Lilja G, Finnbogadóttir H, . Detailed analysis of health-related quality of life after out-of-hospital cardiac arrest. Resuscitation. 2019;135:197-204. doi:10.1016/j.resuscitation.2018.10.028 30385386

[17] Viktorisson A, Sunnerhagen KS, Johansson D, Herlitz J, Axelsson Å. One-year longitudinal study of psychological distress and self-assessed health in survivors of out-of-hospital cardiac arrest. BMJ Open. 2019;9(7):e029756. doi:10.1136/bmjopen-2019-029756 31272987 PMC6615909

[18] Larsson K, Hjelm C, Lilja G, Strömberg A, Årestedt K. Differences in self-reported health between cardiac arrest survivors with good cerebral performance and survivors with moderate cerebral disability: a nationwide register study. BMJ Open. 2022;12(7):e058945. doi:10.1136/bmjopen-2021-058945 35820755 PMC9274516

[19] Andrew E, Nehme Z, Bernard S, Smith K. The influence of comorbidity on survival and long-term outcomes after out-of-hospital cardiac arrest. Resuscitation. 2017;110:42-47. doi:10.1016/j.resuscitation.2016.10.018 27816529

[20] Israelsson J, Thylén I, Strömberg A, Bremer A, Årestedt K. Factors associated with health-related quality of life among cardiac arrest survivors treated with an implantable cardioverter-defibrillator. Resuscitation. 2018;132:78-84. doi:10.1016/j.resuscitation.2018.09.002 30201535

[21] Dillenbeck E, Svensson L, Rawshani A, . Neurologic recovery at discharge and long-term survival after cardiac arrest. JAMA Netw Open. 2024;7(10):e2439196. doi:10.1001/jamanetworkopen.2024.39196 39392629 PMC11581594

[22] Andrew E, Nehme Z, Wolfe R, Bernard S, Smith K. Long-term survival following out-of-hospital cardiac arrest. Heart. 2017;103(14):1104-1110. doi:10.1136/heartjnl-2016-310485 28258247

[23] Chan PS, McNally B, Nallamothu BK, . Long-term outcomes among elderly survivors of out-of-hospital cardiac arrest. J Am Heart Assoc. 2016;5(3):e002924. doi:10.1161/JAHA.115.002924 27068632 PMC4943267

[24] Doherty Z, Fletcher J, Fuzzard K, Kippen R, Knott C, O’Sullivan B. Short and long-term survival following an in-hospital cardiac arrest in a regional hospital cohort. Resuscitation. 2019;143:134-141. doi:10.1016/j.resuscitation.2019.08.028 31470101

[25] Chan PS, Nallamothu BK, Krumholz HM, ; American Heart Association Get with the Guidelines–Resuscitation Investigators. Long-term outcomes in elderly survivors of in-hospital cardiac arrest. N Engl J Med. 2013;368(11):1019-1026. doi:10.1056/NEJMoa1200657 23484828 PMC3652256

[26] Dumas F, Rea TD. Long-term prognosis following resuscitation from out-of-hospital cardiac arrest: role of aetiology and presenting arrest rhythm. Resuscitation. 2012;83(8):1001-1005. doi:10.1016/j.resuscitation.2012.01.029 22306255

[27] Majewski D, Ball S, Bailey P, Bray J, Finn J. Long-term survival among OHCA patients who survive to 30 days: does initial arrest rhythm remain a prognostic determinant? Resuscitation. 2021;162:128-134. doi:10.1016/j.resuscitation.2021.02.030 33640430

[28] Memar M, Geara SJ, Hjalmarsson P, Allberg A, Bouzereau M, Djärv T. Long-term mortality and morbidity among 30-day survivors after in-hospital cardiac arrests—a Swedish cohort study. Resuscitation. 2018;124:76-79. doi:10.1016/j.resuscitation.2018.01.003 29309881

[29] Geri G, Fahrenbruch C, Meischke H, . Effects of bystander CPR following out-of-hospital cardiac arrest on hospital costs and long-term survival. Resuscitation. 2017;115:129-134. doi:10.1016/j.resuscitation.2017.04.016 28427882

[30] Nagy KV, Széplaki G, Perge P, . Quality of life measured with EuroQol-five dimensions questionnaire predicts long-term mortality, response, and reverse remodelling in cardiac resynchronization therapy patients. Europace. 2018;20(9):1506-1512. doi:10.1093/europace/eux342 29182734 PMC6123937

[31] Pocock S, Brieger DB, Owen R, . Health-related quality of life 1-3 years post-myocardial infarction: its impact on prognosis. Open Heart. 2021;8(1):e001499. doi:10.1136/openhrt-2020-001499 33563776 PMC7962722

[32] Lenzen MJ, Scholte op Reimer WJ, Pedersen SS, ; Euro Heart Survey on Coronary Revascularization. The additional value of patient-reported health status in predicting 1-year mortality after invasive coronary procedures: a report from the Euro Heart Survey on Coronary Revascularisation. Heart. 2007;93(3):339-344. doi:10.1136/hrt.2005.086868 16980515 PMC1861429

[33] Lee J, Cho Y, Oh J, . Analysis of anxiety or depression and long-term mortality among survivors of out-of-hospital cardiac arrest. JAMA Netw Open. 2023;6(4):e237809. doi:10.1001/jamanetworkopen.2023.7809 37043200 PMC10098954

[34] Statistics Sweden. Folkmängden efter region, civilstånd, ålder och kön. År 1968-2021. Accessed December 9, 2022. https://www.statistikdatabasen.scb.se/pxweb/sv/ssd/START__BE__BE0101__BE0101A/BefolkningNy/

[35] Langhelle A, Nolan J, Herlitz J, ; 2003 Utstein Consensus Symposium. Recommended guidelines for reviewing, reporting, and conducting research on post-resuscitation care: the Utstein style. Resuscitation. 2005;66(3):271-283. doi:10.1016/j.resuscitation.2005.06.005 16129543

[36] Svenska hjärt-lungräddningsregistret (Swedish Register for Cardiopulmonary Resuscitation). Svenska Hjärt-Lungräddningsregistret. Årsrapport 2015. Årsrapport 2014 års resultat. Accessed June 2, 2025. https://registercentrum.blob.core.windows.net/shlrsjh/r/-rsrapport-2015-H1BIE_zGZ.pdf

[37] Svenska hjärt-lungräddningsregistret (Swedish Register for Cardiopulmonary Resuscitation). Årsrapport för år 2019. Accessed June 2, 2025. https://arsrapporter.registercentrum.se/shlr/20201103/

[38] Strömsöe A, Svensson L, Axelsson ÅB, Göransson K, Todorova L, Herlitz J. Validity of reported data in the Swedish Cardiac Arrest Register in selected parts in Sweden. Resuscitation. 2013;84(7):952-956. doi:10.1016/j.resuscitation.2012.12.026 23313425

[39] Silverplats J, Äng B, Källestedt MS, Strömsöe A. Incidence and case ascertainment of treated in-hospital cardiac arrest events in a national quality registry—a comparison of reported and non-reported events. Resuscitation. 2024;195:110119. doi:10.1016/j.resuscitation.2024.110119 38244762

[40] Brooke HL, Talbäck M, Hörnblad J, . The Swedish cause of death register. Eur J Epidemiol. 2017;32(9):765-773. doi:10.1007/s10654-017-0316-1 28983736 PMC5662659

[41] National Patient Register. Stockholm 2019 (updated September 4, 2021). Swedish National Board of Health and Welfare. Accessed December 17, 2024. https://www.socialstyrelsen.se/en/statistics-and-data/registers/national-patient-register/

[42] Ludvigsson JF, Svedberg P, Olén O, Bruze G, Neovius M. The longitudinal integrated database for health insurance and labour market studies (LISA) and its use in medical research. Eur J Epidemiol. 2019;34(4):423-437. doi:10.1007/s10654-019-00511-8 30929112 PMC6451717

[43] Ludvigsson JF, Almqvist C, Bonamy AK, . Registers of the Swedish total population and their use in medical research. Eur J Epidemiol. 2016;31(2):125-136. doi:10.1007/s10654-016-0117-y 26769609

[44] Statistics Sweden. Accessed December 17, 2024. https://www.scb.se/en/finding-statistics/

[45] EuroQol Group. EuroQol—a new facility for the measurement of health-related quality of life. Health Policy. 1990;16(3):199-208. doi:10.1016/0168-8510(90)90421-9 10109801

[46] Feng YS, Jiang R, Pickard AS, Kohlmann T. Combining EQ-5D-5L items into a level summary score: demonstrating feasibility using non-parametric item response theory using an international dataset. Qual Life Res. 2022;31(1):11-23. doi:10.1007/s11136-021-02922-1 34236579 PMC8800896

[47] Bohm M, Årestedt K, Ullén S, . Psychometric evaluation of EQ-5D-5L in OHCA survivors from the TTM2 trial: a *post hoc* analysis. Resusc Plus. 2025;24:100994. doi:10.1016/j.resplu.2025.100994 40530409 PMC12173684

[48] Snaith RP. The Hospital Anxiety and Depression Scale. Health Qual Life Outcomes. 2003;1:29. doi:10.1186/1477-7525-1-29 12914662 PMC183845

[49] Grand J, Schiele F, Hassager C, . Quality indicators for post-resuscitation care after out-of-hospital cardiac arrest: a joint statement from the Association for Acute Cardiovascular Care of the European Society of Cardiology, the European Resuscitation Council, the European Society of Intensive Care Medicine, and the European Society for Emergency Medicine. Eur Heart J Acute Cardiovasc Care. 2023;12(3):197-210. 36738295 10.1093/ehjacc/zuad006

[50] Steinberg JS, Joshi S, Schron EB, Powell J, Hallstrom A, McBurnie M; AVID Investigators. Psychosocial status predicts mortality in patients with life-threatening ventricular arrhythmias. Heart Rhythm. 2008;5(3):361-365. doi:10.1016/j.hrthm.2007.11.010 18313592

[51] Lahoud R, Chongthammakun V, Wu Y, Hawwa N, Brennan DM, Cho L. Comparing SF-36® scores versus biomarkers to predict mortality in primary cardiac prevention patients. Eur J Intern Med. 2017;46:47-55. doi:10.1016/j.ejim.2017.05.026 28625611

[52] Pocock S, Bueno H, Licour M, . Predictors of one-year mortality at hospital discharge after acute coronary syndromes: a new risk score from the EPICOR (long-tErm follow uP of antithrombotic management patterns In acute CORonary syndrome patients) study. Eur Heart J Acute Cardiovasc Care. 2015;4(6):509-517. doi:10.1177/2048872614554198 25301783 PMC4657391

[53] Kojima G, Iliffe S, Walters K. Frailty index as a predictor of mortality: a systematic review and meta-analysis. Age Ageing. 2018;47(2):193-200. doi:10.1093/ageing/afx162 29040347

[54] Meyer T, Hussein S, Lange HW, Herrmann-Lingen C. Anxiety is associated with a reduction in both mortality and major adverse cardiovascular events five years after coronary stenting. Eur J Prev Cardiol. 2015;22(1):75-82. doi:10.1177/2047487313505244 24045768

[55] Celano CM, Millstein RA, Bedoya CA, Healy BC, Roest AM, Huffman JC. Association between anxiety and mortality in patients with coronary artery disease: a meta-analysis. Am Heart J. 2015;170(6):1105-1115. doi:10.1016/j.ahj.2015.09.013 26678632 PMC4684590

[56] Berg SK, Thorup CB, Borregaard B, . Patient-reported outcomes are independent predictors of one-year mortality and cardiac events across cardiac diagnoses: findings from the national DenHeart survey. Eur J Prev Cardiol. 2019;26(6):624-637. doi:10.1177/2047487318769766 29638142

[57] Lilja G, Nilsson G, Nielsen N, . Anxiety and depression among out-of-hospital cardiac arrest survivors. Resuscitation. 2015;97:68-75. doi:10.1016/j.resuscitation.2015.09.389 26433116

[58] Yaow CYL, Teoh SE, Lim WS, . Prevalence of anxiety, depression, and post-traumatic stress disorder after cardiac arrest: a systematic review and meta-analysis. Resuscitation. 2022;170:82-91. doi:10.1016/j.resuscitation.2021.11.023 34826580

