# Error in Figure Keys

**DOI:** 10.1001/jamanetworkopen.2026.0872

**Published:** 2026-02-11

The Original Investigation titled “Health-Related Quality of Life and Long-Term Survival After Cardiac Arrest,”^1^ published on January 7, 2026, was corrected to fix an error in the figure keys for Figure 2; the color representing the EuroQoL 5-Dimension 5-Level Tool level sum score of 5 (LSS 5) was transposed with the color representing a score of 11 to 25 (LSS 11-25). This article was corrected online.^1^
