# Supplementary material for: Health-Related Quality of Life and Long-Term Survival After Cardiac Arrest
Source: JAMA Netw Open. 2026 Jan 7;9(1):e2552832. doi: 10.1001/jamanetworkopen.2025.52832 (PMC12780932; doi:10.1001/jamanetworkopen.2025.52832)
Supplement: Supplement 1. — eFigure 1. Unadjusted survival plot for patients with in-hospital cardiac arrest (IHCA) eFigure 2. Unadjusted survival plot for patients with out-of-hospital cardiac arrest (OHCA) eFigure 3. Unadjusted hazard ratios (HRs) for death during follow-up as functions of EQ-5D-5L level sum score (LSS) and EQ VAS in IHCA and OHCA populations eFigure 4. Unadjusted hazard ratios (HRs) for death during follow-up as functions of Hospital anxiety and depression scale (HADS) anxiety scores and HADS depression scores in IHCA and OHCA populations eFigure 5. Forest plot of adjusted hazards ratios (HRs) for death during study follow-up for each EQ dimension in IHCA and OHCA populations [file jamanetwopen-e2552832-s001.pdf]

Supplementary Online Content

Dillenbeck E, Nordberg P, Awad A, et al. Health-related quality of life and long-term survival after cardiac arrest. *JAMA Netw Open*. 2026;9(1):e2552832.
doi:10.1001/jamanetworkopen.2025.52832

eFigure 1. Unadjusted survival plot for patients with in-hospital cardiac arrest (IHCA)

eFigure 2. Unadjusted survival plot for patients with out-of-hospital cardiac arrest (OHCA)

eFigure 3. Unadjusted hazard ratios (HRs) for death during follow-up as functions of EQ-5D-5L level sum score (LSS) and EQ VAS in IHCA and OHCA populations

eFigure 4. Unadjusted hazard ratios (HRs) for death during follow-up as functions of Hospital anxiety and depression scale (HADS) anxiety scores and HADS depression scores in IHCA and OHCA populations

eFigure 5. Forest plot of adjusted hazards ratios (HRs) for death during study follow-up for each EQ dimension in IHCA and OHCA populations

This supplementary material has been provided by the authors to give readers additional information about their work.

© 2025 Dillenbeck E et al. *JAMA Network Open*.

eFigure 1. Unadjusted survival plot for patients with in-hospital cardiac arrest (IHCA)

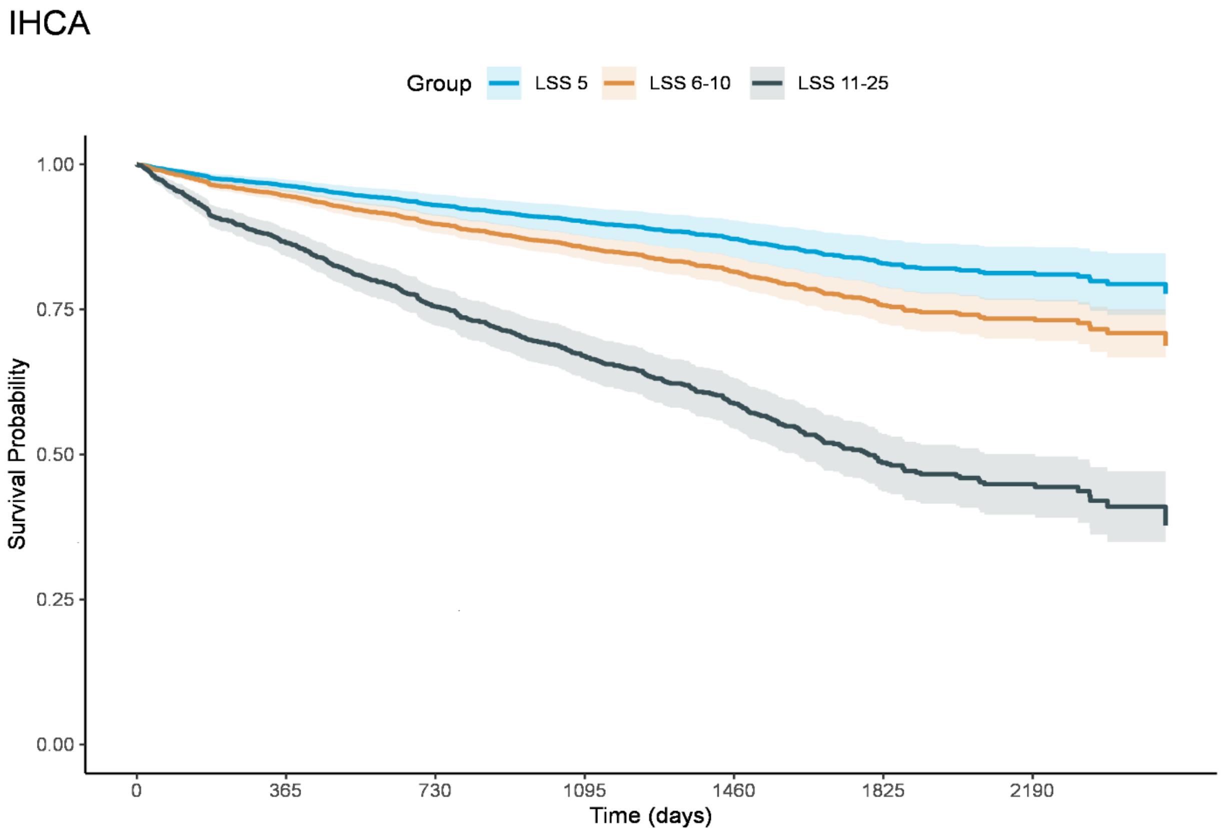

Number at Risk

	0	365	730	1095	1460	1825	2190
LSS 5	394	386	336	253	177	113	46
LSS 6-10	1034	972	802	604	418	237	125
LSS 11-25	572	483	361	255	170	95	36

Time (days)

LSS indicates level sum score

© 2025 Dillenbeck E et al. *JAMA Network Open*.

eFigure 2: Unadjusted survival plot for patients with out-of-hospital cardiac arrest (OHCA)

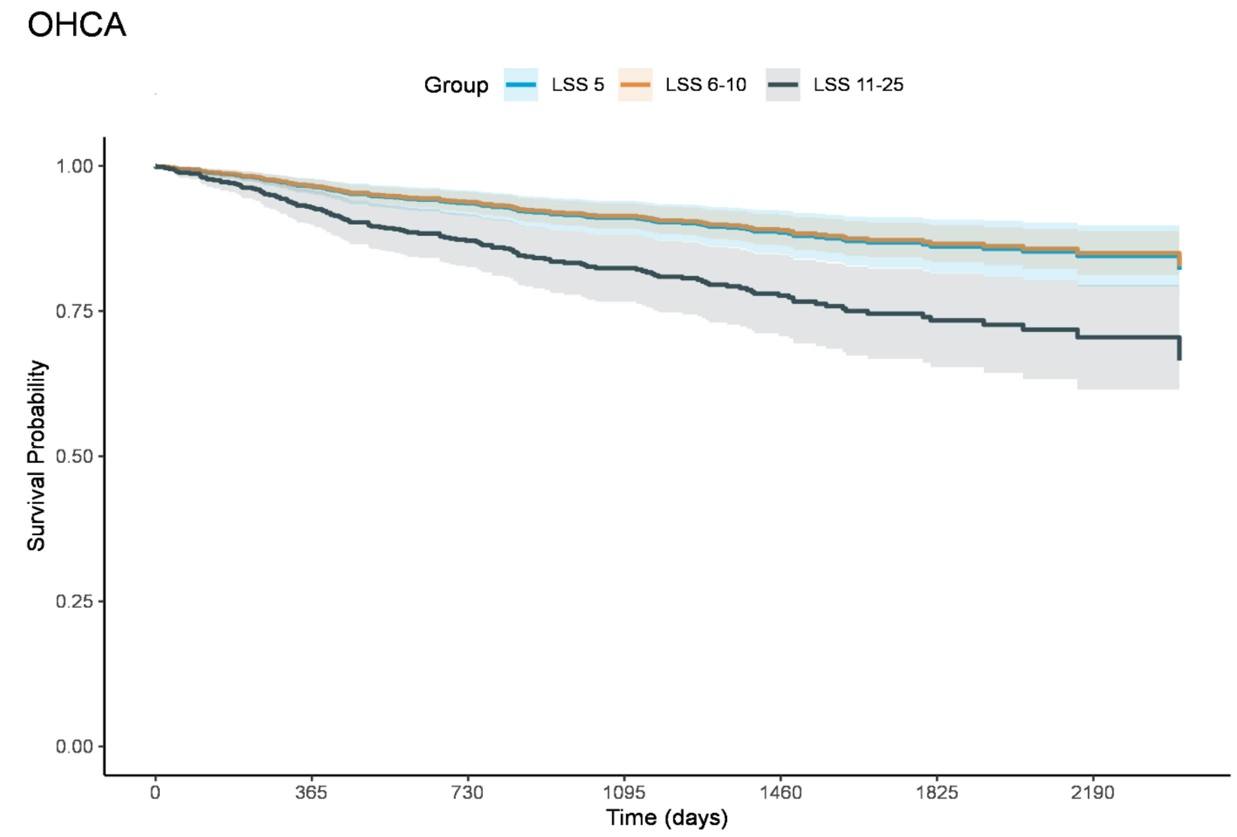

Number at Risk

	0	365	730	1095	1460	1825	2190
LSS 5	301	295	245	185	128	63	24
LSS 6-10	639	612	508	375	251	141	64
LSS 11-25	168	150	115	82	55	22	11

Time (days)

LSS indicates level sum score

© 2025 Dillenbeck E et al. *JAMA Network Open*.

eFigure 3. Unadjusted hazard ratios (HRs) for death during follow-up as functions of EQ-5D-5L level sum score (LSS) and EQ VAS in IHCA and OHCA populations

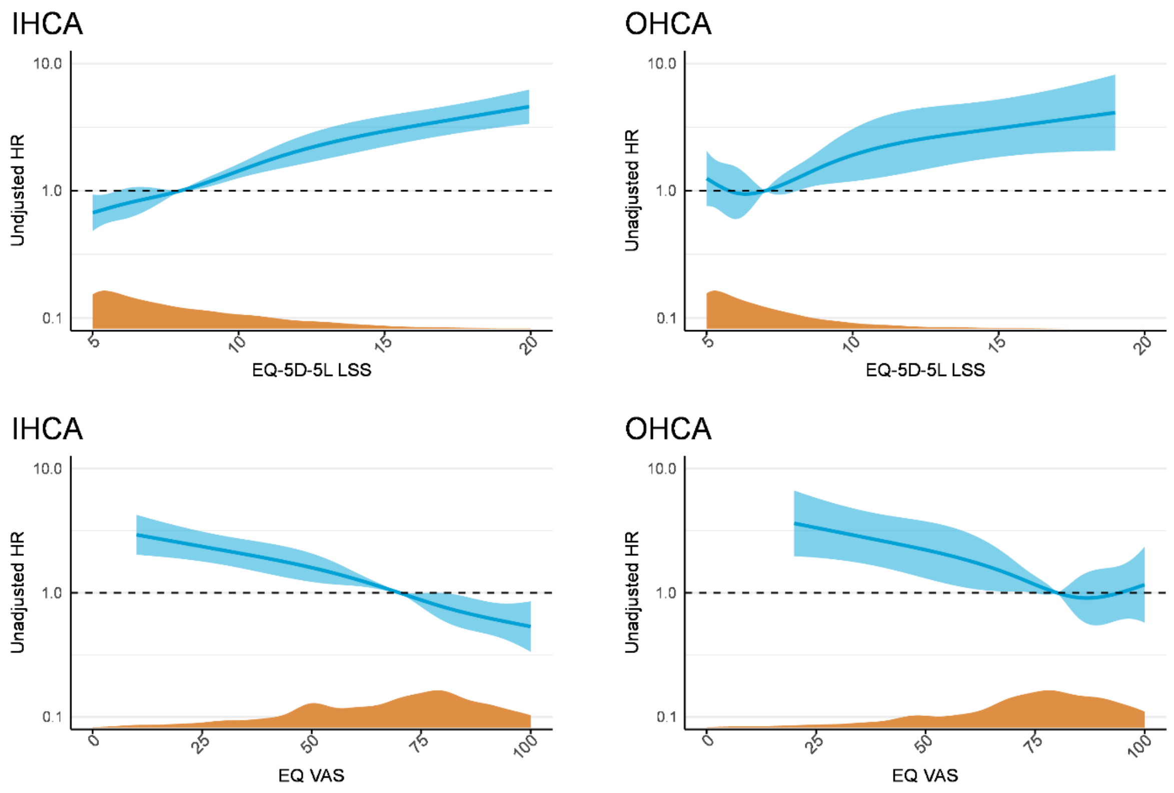

Blue lines represent HRs with 95% confidence intervals shown in light blue. Yellow plots show the density of LSS and EQ VAS, respectively. IHCA indicates in-hospital cardiac arrest; OHCA, out-of-hospital cardiac arrest; VAS, visual analogue scale.

© 2025 Dillenbeck E et al. *JAMA Network Open*.

eFigure 4. Unadjusted hazard ratios (HRs) for death during follow-up as functions of Hospital anxiety and depression scale (HADS) anxiety scores and HADS depression scores in IHCA and OHCA populations

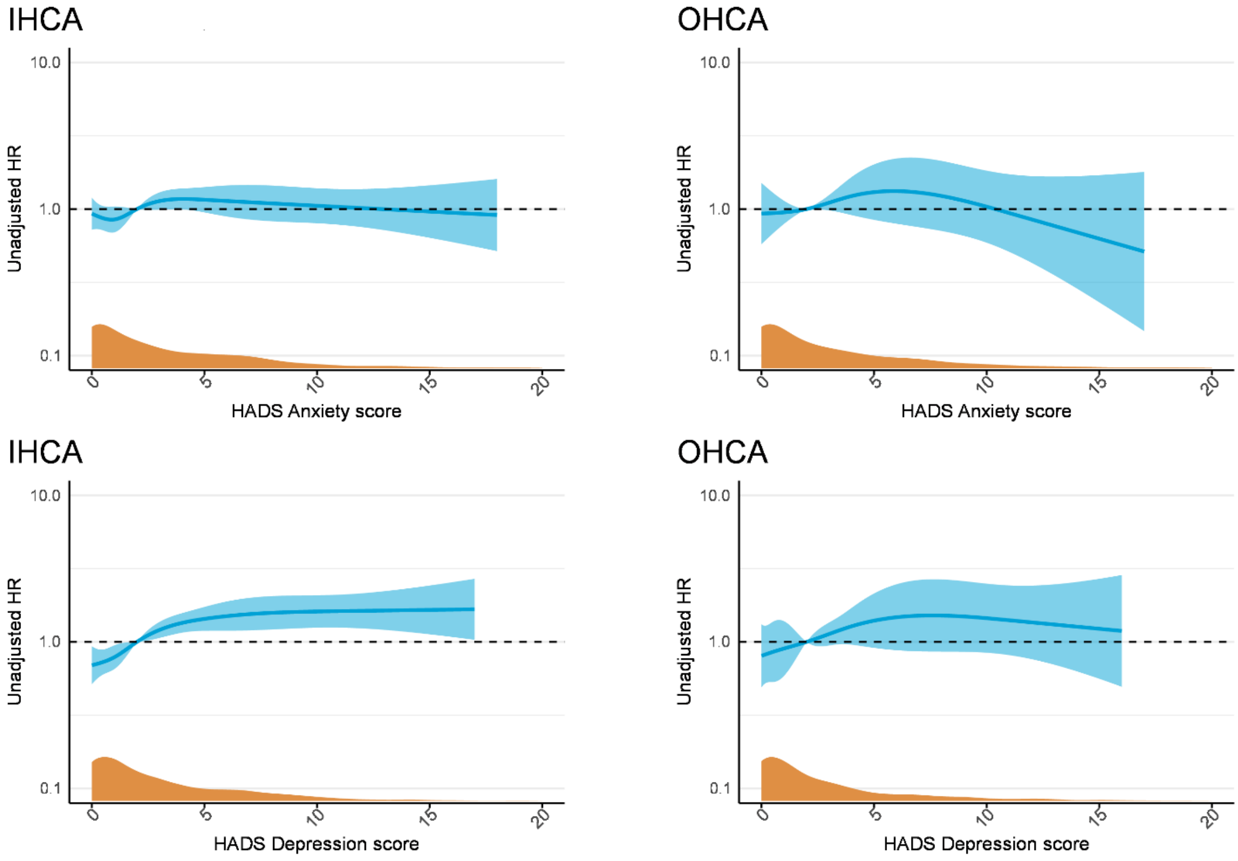

Blue lines represent HRs with 95% confidence intervals shown in light blue. Yellow plots show the density of HADS anxiety scores and HADS depression scores, respectively. IHCA indicates in-hospital cardiac arrest; OHCA, out-of-hospital cardiac arrest.

© 2025 Dillenbeck E et al. *JAMA Network Open*.

eFigure 5. Forest plot of adjusted hazards ratios (HRs) for death during study follow-up for each EQ dimension in IHCA and OHCA populations

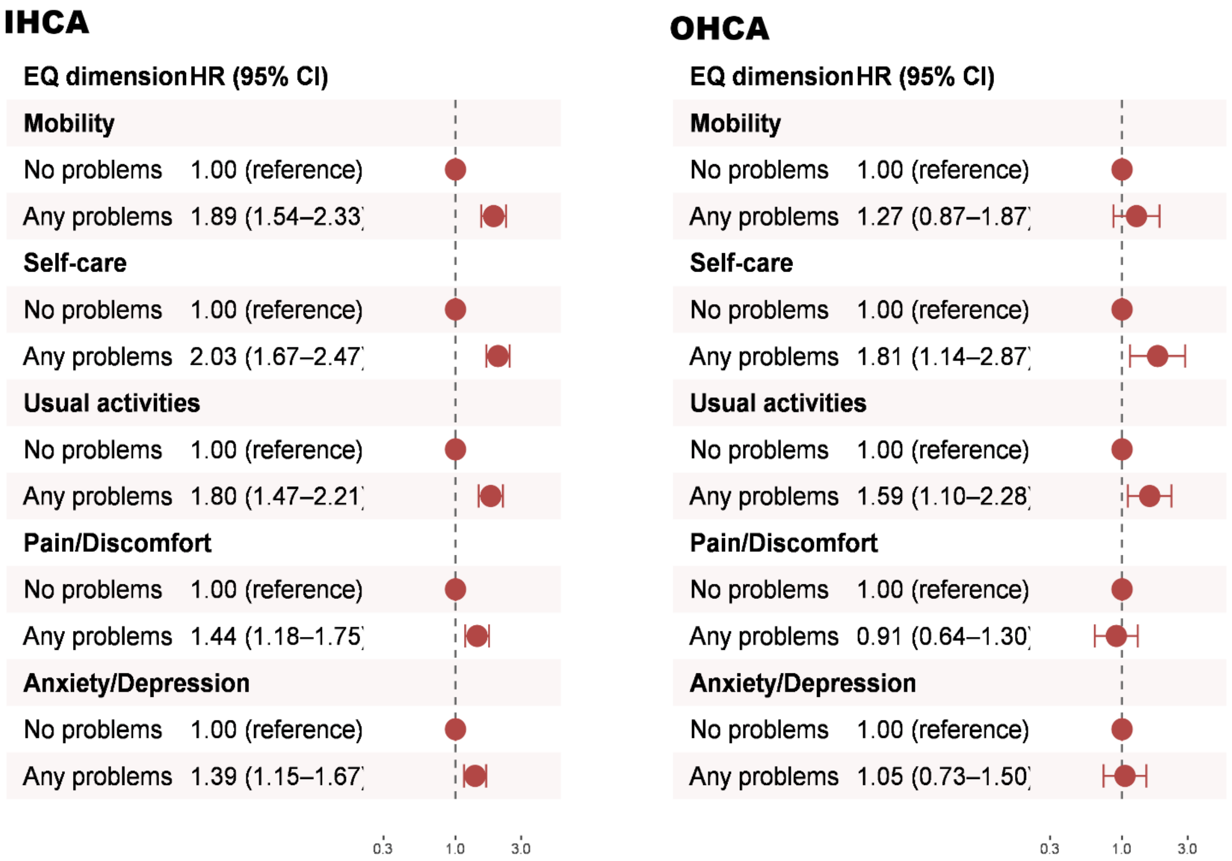

HR indicates hazard ratio; IHCA, in-hospital cardiac arrest; OHCA, out-of-hospital cardiac arrest.

© 2025 Dillenbeck E et al. *JAMA Network Open*.
