# Supplementary material for: Health-Related Quality of Life and Long-Term Survival After Cardiac Arrest
Source: JAMA Netw Open. 2026 Jan 7;9(1):e2552832. doi: 10.1001/jamanetworkopen.2025.52832 (PMC12780932; doi:10.1001/jamanetworkopen.2025.52832)
Supplement: Supplement 2. — Data Sharing Statement [file jamanetwopen-e2552832-s002.pdf]

Data Sharing Statement

Dillenbeck. Health-Related Quality of Life and Long-Term Survival After Cardiac Arrest. *JAMA Netw Open*. Published January 07, 2026. doi:10.1001/jamanetworkopen.2025.52832

Data
Data available: No

Additional Information
Explanation for why data not available: Data cannot be shared due to data protection requirements within national law
